# Spontaneously formed gradient chemical compositional structures of niobium doped titanium dioxide nanoparticles enhance ultraviolet- and visible-light photocatalytic performance

**DOI:** 10.1038/s41598-021-94512-x

**Published:** 2021-07-30

**Authors:** Naoki Tarutani, Ryuma Kato, Tetsuo Uchikoshi, Takamasa Ishigaki

**Affiliations:** 1 Applied Chemistry Program, Graduate School of Advanced Science and Engineering, Hiroshima University, 1-4-1 Kagamiyama, Higashi-Hiroshima, Hiroshima, 739-8527 Japan; 2 Department of Chemical Science and Technology, Faculty of Bioscience and Applied Chemistry, Hosei University, 3-7-2 Kajino-cho, Koganei, Tokyo, 184-8584 Japan; 3 Research Center for Micro-Nano Technology, Hosei University, 3-11-15 Midori-cho, Koganei, Tokyo, 184-0003 Japan; 4 Research Center for Functional Materials, National Institute for Materials Science, 1-2-1, Sengen, Tsukuba, 305-0047 Japan

**Keywords:** Photocatalysis, Nanoparticles, Synthesis and processing

## Abstract

Semiconductor photocatalysts showing excellent performance under irradiation of both ultraviolet (UV)- and visible (VIS)-light are highly demanded towards realization of sustainable energy systems. TiO_2_ is one of the most common photocatalysts and has been widely investigated as candidate showing UV/VIS responsive performance. In this study, we report synthesis of Nb doped TiO_2_ by environmentally benign mechanochemical reaction. Nb atoms were successfully incorporated into TiO_2_ lattice by applying mechanical energy. As synthesized Nb doped TiO_2_ were metastable phase and formed chemical compositional gradient structure of poorly Nb doped TiO_2_ core and highly Nb doped TiO_2_ surface after high temperature heat treatment. It was found that formed gradient chemical compositional heterojunctions effectively enhanced photocatalytic performance of Nb doped TiO_2_ under both of UV- and VIS-light irradiation, which is different trend compared with Nb doped TiO_2_ prepared through conventional methods. The approach shown here will be employed for versatile systems because of simple and environmentally benign process.

## Introduction

In the last decade, there is a numerous number of studies about functionality improvement of semiconductor photocatalysts to achieve both of ultraviolet (UV)-light and visible (VIS)-light responsive performance. Several strategies, such as band gap engineering by doping cations and/or anions, modification of surface structures, and design heterojunction with cocatalysts of metals or semiconductors, were employed to achieve UV/VIS responsive photocatalytic performance^1–4^. Although typical methods, such as solid state reaction^5,6^, solvothermal/hydrothermal reaction^7–10^, and vacuum deposition^11,12^, are successful approach to synthesize doped semiconductors and heterojunction materials, it is necessary to employ energy consuming and/or high environmental burden conditions, such as quite high temperature sintering, toxic gas flow, high pressure, and vacuum condition, which will give negative impact on environment. Mechanochemical process is known as a facile and an environmentally benign method^13,14^. Mechanochemical reaction often produces unique metastable materials which cannot be prepared by conventional thermochemical techniques^15^. For example, metastable amorphous calcium carbonate phase was successfully stabilized by mechanochemical Na doping^16^. Synthesis of metastable Li_2_OHCl is another example, which showed higher ionic conductivity compared with thermodynamically stable phase^17^. Nowadays, mechanochemical process is widely employed to synthesize not only typical organic and inorganic compounds, but also doped compounds and composites^18–21^. Moreover, the mechanisms of the co-crystallization and metastable formation through mechanochemical reactions were reported in the specific systems^22,23^. There is a strong demand to develop facile and promising process by using mechanochemical reaction for highly functional materials, such as UV- and VIS-light responsive semiconductor photocatalysts.

Titanium dioxide, TiO_2_, is one of the most investigated semiconductor which shows excellent photocatalytic activity under irradiation of UV-light^24^. Doping of metals/nonmetals and/or formation of heterojunction of TiO_2_/semiconductors were reported to effectively enhance photocatalytic performance^25,26^. For a preparation of doped TiO_2_ through mechanochemical reaction, metals and metal salts were used as dopant sources of cations in general. However, metals tend to weld each other to form composite rather than doping and metal salts gave unexpected doping from counter anions^18,27^. To avoid these drawbacks, it is necessary to optimize numerous mechanochemical reaction parameters, such as gas atmosphere, milling media, milling speed, milling time, and ball-to-powder weight ratio. Use of metal oxides instead of metals and metal salts is expected to be easier and successful way to dope metal cations into TiO_2_ matrixes.

In this study, we focused on the fabrication of Nb doped TiO_2_ by using mechanochemical process. Commercially available TiO_2_ nanoparticles (mixture of anatase and rutile phase) was employed as a starting material and synthesized TiNb_2_O_7_ microparticles was used as a Nb doping agent. Variety amounts of Nb atom were homogeneously incorporated into both of anatase and rutile nanoparticles by wet mechanochemical process. Nb doped anatase nanoparticles showed improved thermal stability owing to the pinning Nb atoms. It was found that heat treatment led Nb atoms distributed inhomogeneously at surface of nanoparticles to form gradient chemical compositional structures of poorly Nb doped TiO_2_ and highly Nb doped TiO_2_. The Nb doped TiO_2_ with chemical compositional gradient structure showed enhanced photocatalytic activity under both of UV- and VIS-light irradiation. Although Nb doped TiO_2_ were synthesized through vairous methods, such as pulsed laser deposition^28,29^, sol–gel technique^30,31^, thermal plasma processing^32^, and spray pyrolysis^33^, chemical compositional gradient structures have not been reported. This indicates that mechanochemically synthesized materials are key to form the gradient structure and enhance photocatalytic property. The method reported here is a simple and facile approach, hence it is expected to widely apply other dopant-semiconductor systems towards highly functionalized photocatalysts.

## Methods

### Chemicals

Titanium(IV) tetraisopropoxide (TTIP, 95%), niobium(V) pentaethoxide (NPE, 99.9%), diethanolamine (99%), polyethyleneimine (*M*_w_ = 1800), methyl orange (MO), sodium dihydrogen phosphate (NaH_2_PO_4_, 85%), phosphoric acid (H_3_PO_4_, 85%), ethanol (99.5%) were used as received. H_3_PO_4_ was purchased from Kanto Chemical Holdings. All other reagents were purchased from Wako Pure Chemicals Industries, Ltd. Ultrapure water of 18.2 MΩ∙cm resistivity was used in all experiments.

### Synthesis of TiNb_2_O_7_ microparticles

TTIP (1 mmol), NPE (2 mmol), and diethanolamine (2 mmol) were dissolved in 5 mL of ethanol. After stirring for 20 min, homogenous sol was obtained. Water (35 mmol) was added to the sol under vigorous stirring and keep stirring at least for 5 min to obtain gel. Gelatinous precipitates were dried at 100 °C for 12 h. Dried powders were heat treated at 1150 °C for 3 h (ramp rate: 5 °C/min). Heat treated powder of TiNb_2_O_7_ was employed as a Nb source in subsequent mechanochemical process because TiNb_2_O_7_ was reportedly segregated from Nb doped TiO_2_ only after high temperature heat treatment that means TiNb_2_O_7_ will be highly compatible with TiO_2_^32^.

### Mechanochemical synthesis of Nb-doped TiO_2_ nanoparticles

Obtained TiNb_2_O_7_ powders were mixed with commercial anatase–rutile TiO_2_ nanoparticles (EVONIK AEROXIDE® P25, Nippon Aerosil, Tokyo, Japan) with a molar ratio of Nb/(Ti + Nb) = 0 (without TiNb_2_O_7_), 0.02, 0.05, and 0.10. After grounded for 30 min in a mortar, mixed powders (2.5 g) were dispersed in 20 mL of ethanol solution including polyethyleneimine (7.2 × 10^−2^ mmol). Obtained suspensions were sealed in zirconia pod (inner volume of 80 mL) and milled in a tilted rotation planetary ball mill (Planet M2-3F, Nagao System Inc.) at revolution speed of 700 rpm and rotation speed of 1750 rpm (152 G) for 2 h with zirconia balls (5 mm in diameter, #243, 83.5 g). Milled suspensions were dried at room temperature for 5 days and heat treated at 500 − 900 °C for 3 h (ramp rate: 5 °C/min, cooling rate: 2 °C/min). The powders prepared through this process were denoted as NTO-*x*-*y*, where *x* and *y* are nominal Nb/(Ti + Nb) in percentage (0 ≤ *x* ≤ 10) and heat treatment temperature (500 ≤ *y* ≤ 900) (As dried samples before heat treatment are denoted as NTO-*x*-dry).

### Photocatalytic test

The solution pH was kept at 2.1 using buffer solution to avoid agglomeration of nanoparticles during photocatalytic test, which changes background light scattering by particles. Phosphate buffer solution (0.10 mol/L, pH = 2.1) were prepared using NaH_2_PO_4_ and H_3_PO_4_. Sample powders (10 mg) were dispersed in 6 mL of buffered aqueous solution and ultrasonicated for 30 min. MO buffered aqueous solution (20 μmol/L, 20 μL) was added to the suspensions. Colored suspensions (3 mL) in close capped quartz cell (1 × 1 cm^2^ base rectangular cell) were exposed to UV/VIS-light after stirring in dark condition. UV light and VIS light were generated by UVF-203S Type-A light source (San-Ei Electric Co., Ltd., Japan) (365 nm) and UVF–203S Type-C light source (San-Ei Electric Co., Ltd., Japan) (405 and 436 nm) with intensities of 60 and 255 mW/cm^2^, respectively. The light probes were contacted to the quartz cell, which means the light-to-solution distance was same for all the experiments, ~ 1 mm. After appropriate irradiation time, the suspensions were left for 30 s without stirring and set in a UV–vis spectroscopy to determine MO concentration from the *F*(R_∞_) at 514 nm of diffuse reflectance spectra using calibration curve.

### Characterization

A field emission scanning microscope (SEM; S-8020, Hitachi High-Technologies Corp., Japan) equipped with energy dispersed X-ray spectroscopy (EDS) was used for evaluation of particle size and elemental composition. A transmission electron microscope (TEM; JEM-2100F, JEOL Ltd., Japan) equipped with EDS and scanning TEM mode (STEM) was employed at an operating voltage of 200 kV to clarify the crystal phase and elemental composition of individual particles. Powder X-ray diffraction (XRD) (SmartLab, Rigaku Corp., Japan) using Cu K*α* radiation (*λ* = 0.1544 nm) equipped with one dimensional detector was used to characterize crystal phases of samples. X-ray photoelectron spectrometer (XPS) analyses were carried out with ESCA-5600 (Ulvac-Phi, Japan) using a monochromatic Al K*α* source (1486.6 eV, 200 W) to evaluate an elemental composition of adjacent surface of particles and an oxidation state of Ti and Nb atoms. The instrument work function was calibrated to give an Au 4f_7/2_ of metallic gold binding energy of 83.95 eV ± 0.1 eV. Survey spectra and high-resolution spectra were collected with a step of 0.4 and 0.05 eV, respectively. The obtained spectra were calibrated using C 1 s binding energy (284.8 eV) of the samples as an internal standard. Ultraviolet–visible (UV–Vis) spectroscopy (V-650 spectrophotometer, JASCO Corp., Japan) equipped with an integrating sphere was used to characterize optical band gap of samples and evaluation of decolorization of MO solution. The reflectance values of obtained diffuse reflectance spectra were transformed to Kubelka–Munk units, *F*(*R*_∞_) by using Eq. (1)

1 $$F(R_{\infty } ) = { (}1 - F(R_{{\infty ,{\text{s}}}} ){)}^{2} /2(F(R_{{\infty ,{\text{s}}}} ))$$

where $F(R_{{\infty ,{\text{s}}}} )$ is measured reflectance value. Raman spectroscopy (RAMANtouch, Nanophoton Corp., Japan) was employed to characterize crystal phases and defects (excitation: 532 nm). The N_2_ adsorption–desorption measurement using Belsorp-18 (Bel Japan Inc., Japan) was employed to evaluate specific surface area by the Brunauer–Emmett–Teller (BET) method.

## Results and discussion

### Incorporation of Nb into TiO_2_ nanoparticles through mechanochemical treatment

**Figure 1:**
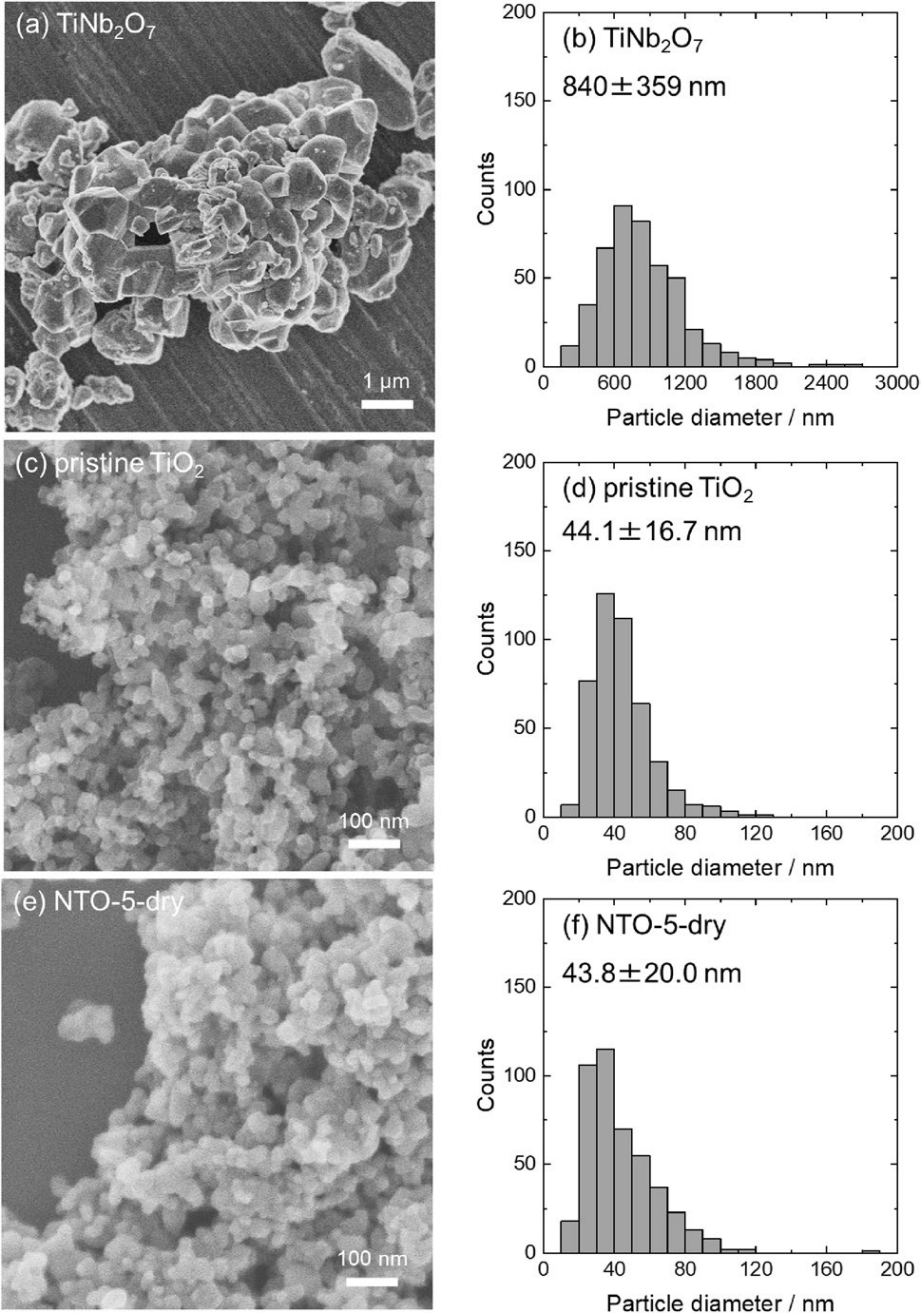
(**a**), (**c**), (**e**) SEM images and (**b**), (**d**), (**f**) corresponding particle diameter distributions (statistics of 450 particles) of synthesized TiNb_2_O_7_ microparticles, pristine TiO_2_ nanoparticles, and NTO-5-dry.

**Figure 2:**
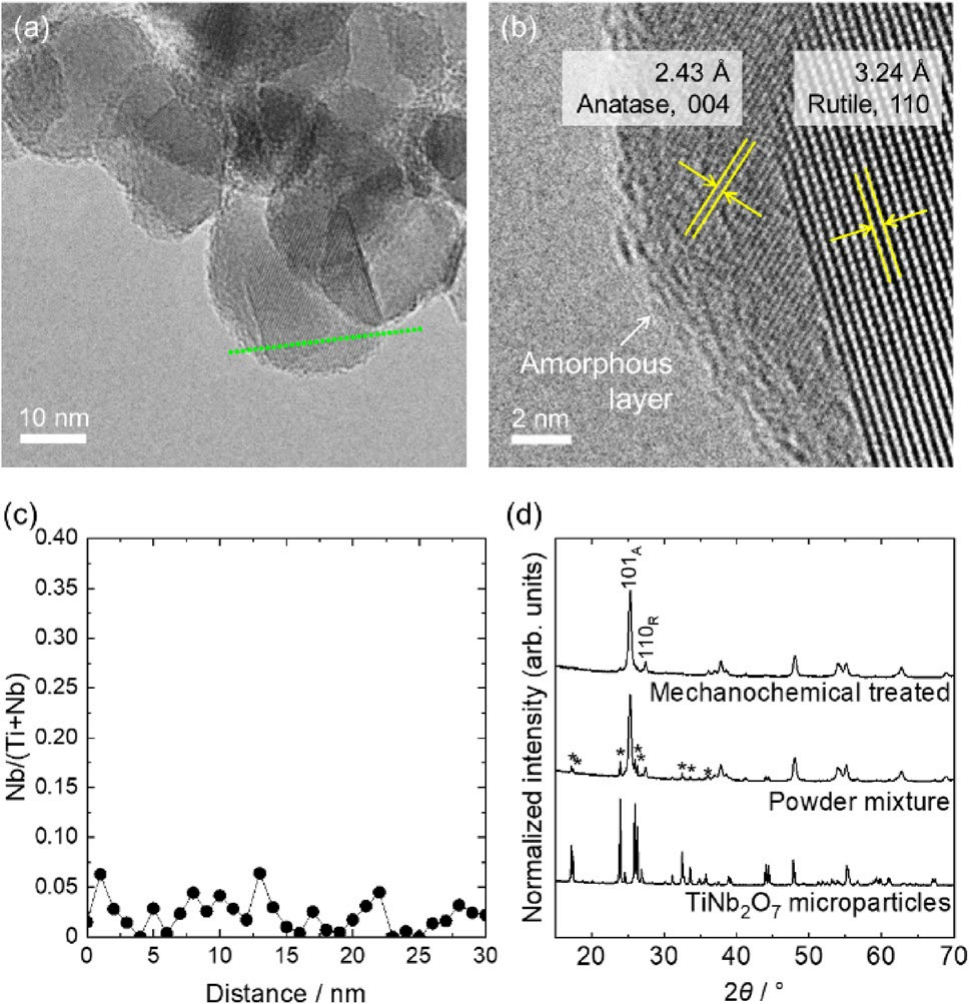
(**a**,**b**) TEM images and (**c**) Nb/(Ti + Nb) calculated from STEM-EDS line scan of NTO-5-dry. Green dots in (**a**) are scanned points of STEM-EDS shown in (**c**). (**d**) XRD patterns of TiNb_2_O_7_ microparticles, powder mixture of pristine TiO_2_ and TiNb_2_O_7_ and mechanochemical treated powder (nominal Nb/(Ti + Nb) = 0.05). * TiNb_2_O_7_; JCPDS #00–072-0116.

TTIP and NPE were used as precursors to synthesize TiNb_2_O_7_ as a Nb doping agent. Addition of water to ethanolic solution, including TTIP, NPE, and diethanolamine, triggered hydrolysis and condensation of both metal alkoxides. During reaction, diethanolamine worked as a reaction controlling agent, which hinder vigorous reaction of NPE and enabled to form homogenous Ti and Nb mixed precipitates. Dried precipitates were characterized as poorly crystalline TiNb_2_O_7_ by XRD measurement (Figure S1). The precipitates were heat treated at 1150 °C for 3 h. Heat treated precipitates were characterized as well-crystalline TiNb_2_O_7_ with a crystallite size of 63.7 nm calculated using 110 diffraction peak. Obtained TiNb_2_O_7_ showed angulate shape with a particle size of 840 ± 359 nm (Fig. 1a,d). TiNb_2_O_7_ microparticles and commercially available TiO_2_ nanoparticles (particle diameter of 44.1 ± 16.7 nm) (Fig. 1b,e) were mixed and mechanochemically treated. After mechanochemical treatment, as dried powder (NTO-5-dry, see experimental section for sample IDs) showed the particle size of 43.8 ± 20.0 nm which is comparable size to the pristine TiO_2_ (Fig. 1c,f). Figure 2a,b are TEM image of NTO-5-dry. The lattice fringe close to particle surface became disorder after mechanochemical treatment, which is a feature of amorphous phase. Raman spectra of pristine TiO_2_, NTO-5-dry, and NTO-5-600 were shown in Figure S2. The peak around 145 cm^−1^, assigned as E_g_ mode of symmetric stretching vibrations of O–Ti–O bonds of anatase phase^34^, was shifted to 148 cm^−1^ after mechanochemical treatment (NTO-5-dry), which indicates formation of oxygen vacancies^13^. Grayish color of NTO-5-dry powder agrees formation of oxygen vacancies in TiO_2_ matrix^35^. The peak shifted back to 145 cm^−1^ after heat treatment at 600 °C indicating elimination of vacancies. The atomic ratios of Nb/(Ti + Nb) evaluated by SEM–EDS and XPS were well matched with nominal molar ratio (Table S1). Figure 2c shows the local Nb/(Ti + Nb) ratio measured by STEM-EDS line scanning. The scanned line was shown in Fig. 2a as green dots, which crosses both of rutile and anatase nanoparticles. Nb atoms were detected at entire points, which implies that Nb atoms were homogeneously incorporated in both of anatase and rutile nanoparticles.

XRD patterns of powders before and after mechanochemical treatment were shown in Fig. 2d with the pattern of TiNb_2_O_7_ microparticles. Detected crystalline species were anatase and rutile phase with trace amount of TiNb_2_O_7_ after mechanochemical reaction. Crystallite size of TiNb_2_O_7_, anatase, and rutile before/after mechanochemical treatment were calculated as 63.7/31.7 nm, 21.4/20.7 nm, and 29.2/25.8 nm, respectively. Drastic crystallite size reduction of TiNb_2_O_7_ indicates that TiO_2_ crystals were more stable than TiNb_2_O_7_ crystals against mechanical energy. Reported Young’s modulus of each crystals (255.5–311.6 GPa for TiO_2_^36^ and 173 ± 5 GPa for TiNb_2_O_7_^37^) supports that preferential amorphization of TiNb_2_O_7_ crystals took place during mechanochemical treatment. Lattice volume of anatase (135.98(8) Å^3^) and rutile (62.48(3) Å^3^) phases of NTO-5-dry was comparable to those of pristine TiO_2_ (136.01(7) Å^3^ and 62.51(3) Å^3^). This may be result of competitive effects between lattice expansion due to Nb substitution^31,32^ and lattice contraction due to interstitial oxygen in TiO_2_^38^ incorporated to compensate charge balance^39,40^.

**Figure 3:**
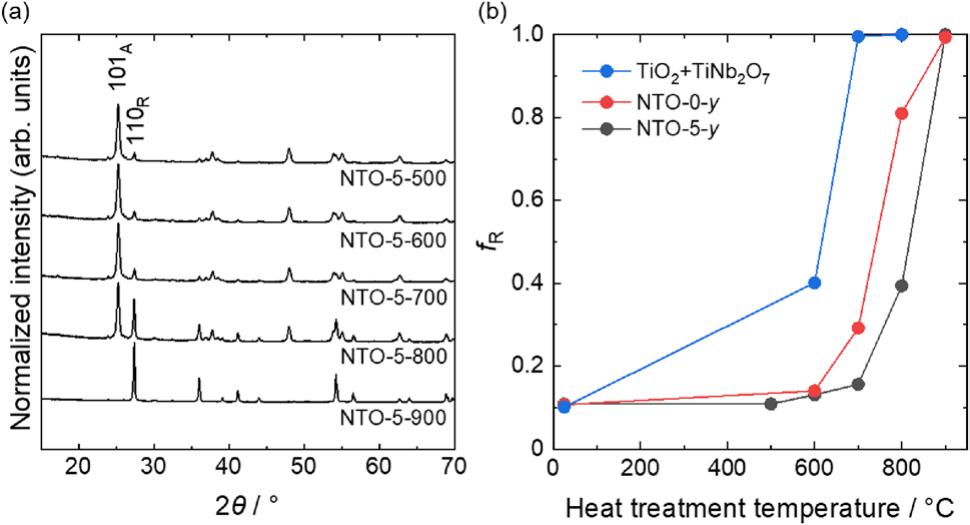
(**a**) XRD patterns of NTO-5-*y* (*y* = 500 − 900). 101_A_ and 110_R_ correspond to 101 diffraction peak of anatase and 110 diffraction peak of rutile, respectively. (**b**) Rutile phase weight fraction, *f*_R_, against anatase phase calculated from corresponding XRD patterns. As mixed pristine TiO_2_ and TiNb_2_O_7_, NTO-0-dry, and NTO-5-dry are plotted at temperature of 25 °C.

Figure 3a shows XRD patterns of powders heat treated at 500–900 °C (NTO-5-*y*, *y* = 500–900). The intensity of peaks assigned as rutile phase increased with increasing the heat treatment temperature. The weight fraction of rutile phase against anatase phase, *f*_R_, was calculated using following Eq. (2)^41^;

2 $$f_{{\text{R}}} = \, 1/\left( {1 + 0.79 \cdot I_{{\text{A}}} /I_{{\text{R}}} } \right)$$

where *I*_A_ and *I*_R_ are integrated intensities of rutile 110 diffraction peak and anatase 101 diffraction peak, respectively. Temperature dependent changes of *f*_R_ were shown in Fig. 3b. NTO-0-dry and NTO-5-dry showed *f*_R_ comparable to pristine TiO_2_. Although a phase transition from anatase to rutile by mechanochemical treatment was reported^42^, amorphous layer formation on the surface of nanoparticles and preferential amorphization of TiNb_2_O_7_ may consume mechanical energy and retard phase transition of anatase. In the case of ground mixed powders of pristine TiO_2_ and TiNb_2_O_7_ in a mortar, *f*_R_ drastically increased with temperature and reached *f*_R_ ~ 1.0 after heat treatment at 700 °C. NTO-0-*y* showed smaller *f*_R_ than powder mixtures at each heat treatment temperature, which indicates formed surface amorphous layer worked as effective grain boundary and retarded phase transition. In the case of NTO-5-*y*, ~ 60% of anatase crystals were remained even after heat treated at 800 °C. In addition to amorphous layer formation, incorporated Nb atoms worked as pinning sites and improved thermal stability^31,32^. Thermal stability of anatase phase was also improved in the case of NTO-2-*y* and NTO-10-*y* (Figure S3a), which indicates that incorporated Nb atoms effectively worked as pining sites even if small quantity.

**Figure 4:**
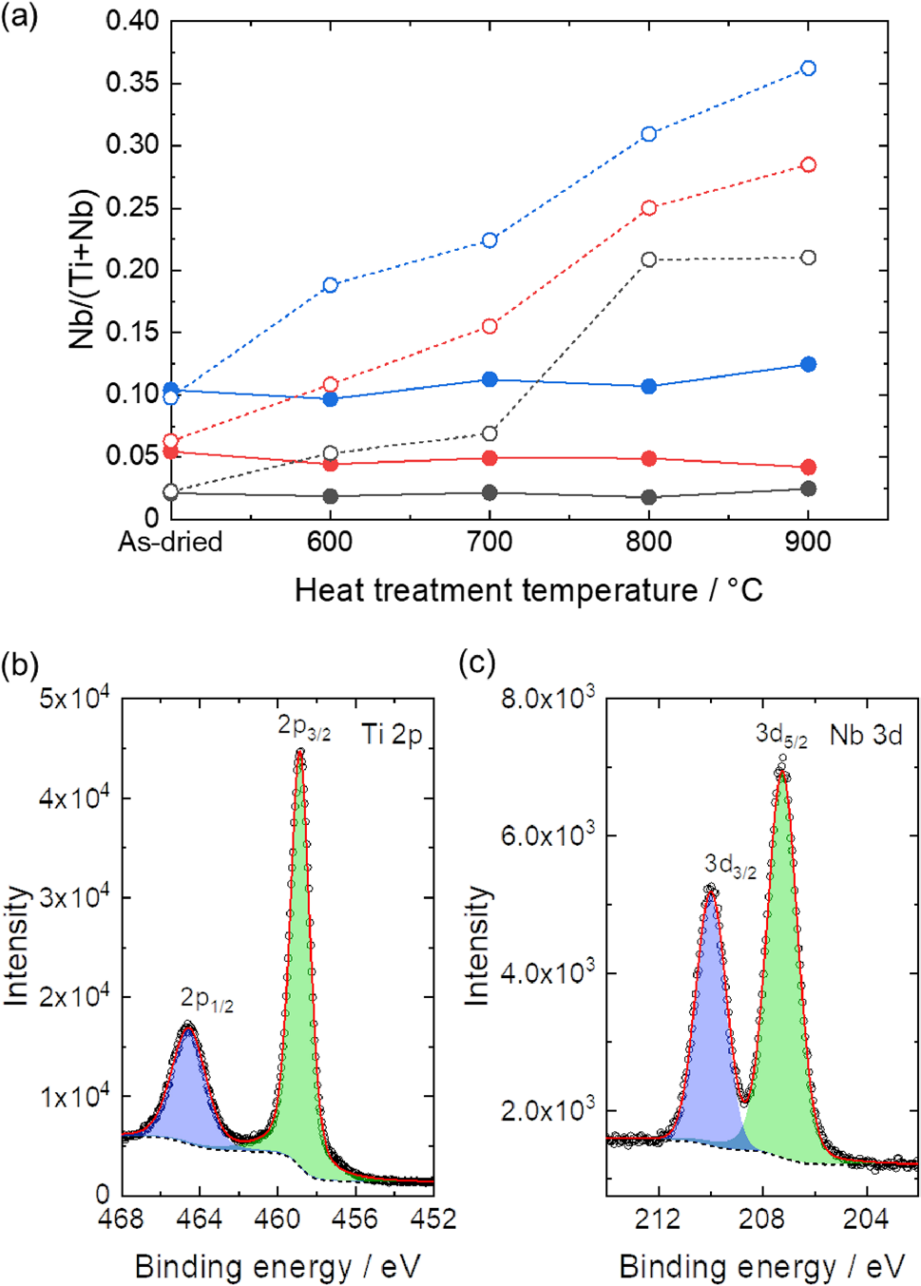
(**a**) Nb/(Ti + Nb) values calculated from SEM–EDS (filled symbol with solid lines) and XPS (open symbol with dot lines) spectra of NTO-2-*y* (black), NTO-5-*y* (red) and NTO-10-*y* (blue). (**b**) Ti 2p and (**c**) Nb 3d XPS spectra of NTO-5–600 (open circles, black dot line, blue/green area, and red solid line denote experimental data, background, fitted curves, and cumulative curves of fitted curves.

Chemical compositions of heat-treated powders were investigated by using SEM–EDS and XPS techniques (Fig. 4a). Nb/(Ti + Nb) ratio calculated from SEM–EDS spectra of NTO-2-*y*, NTO-5-*y*, and NTO-10-*y* were well matched with nominal value. On the other hand, Nb/(Ti + Nb) ratio calculated using XPS spectra were increased with increasing the heat treatment temperature, which means that heat treated Nb doped TiO_2_ have inhomogeneous chemical composition between surface and core of the particles. The Nb/(Ti + Nb) increased with increasing heat treatment temperature independently of the integrated area of diffraction peak of TiNb_2_O_7_, except NTO-10-900 (Figure S3b). This implies that Nb atoms were still incorporated in the TiO_2_ lattice and/or in amorphous like Nb-rich oxide phases at the surface of nanoparticles. Figure 4b,c were XPS Ti 2p and Nb 3d core level spectrum of NTO-5-600. Both spectra were fitted with two of peak functions. The fitted peak positions of Ti 2p_1/2_ and Ti 2p_3/2_ were 464.6 eV and 458.9 eV, respectively, which is a good agreement with reported value of Ti^4+^^43^. The fitted peak positions of Nb 3d_3/2_ and Nb 3d_5/2_ were 210.0 eV and 207.2 eV, respectively. The Nb 3d_5/2_ peak position is intermediate of Nb^4+^ (205.9 eV) and Nb^5+^ (207.5 eV)^44^. In the case of titanium niobium oxides family (TiNb_*m*_O_2+2.5* m*_), relatively Ti rich compounds, such as TiNb_2_O_7_ (*m* = 2)^45^ and Ti_2_Nb_10_O_29_ (*m* = 5)^46^ showed Nb 3d_5/2_ peak position same as mixture of Nb^4+^ and Nb^5+^ in Nb-doped TiO_2_^31^ and relatively Nb rich compounds, such as TiNb_6_O_17_ (*m* = 6)^47^ and TiNb_24_O_62_ (*m* = 24)^48^ showed the peak position same as Nb^5+^ in Nb_2_O_5_. Considering Nb/(Ti + Nb) of NTO-5–600 was 0.11, it is reliable that Nb was exist as a mixture of Nb^4+^ and Nb^5+^. All the samples showed Ti 2p_3/2_ = 458.8 ± 0.2 eV and Nb 3d_5/2_ = 207.1 ± 0.2 eV which indicate that the oxidation states of Ti and Nb were stable during heat treatment.

**Figure 5:**
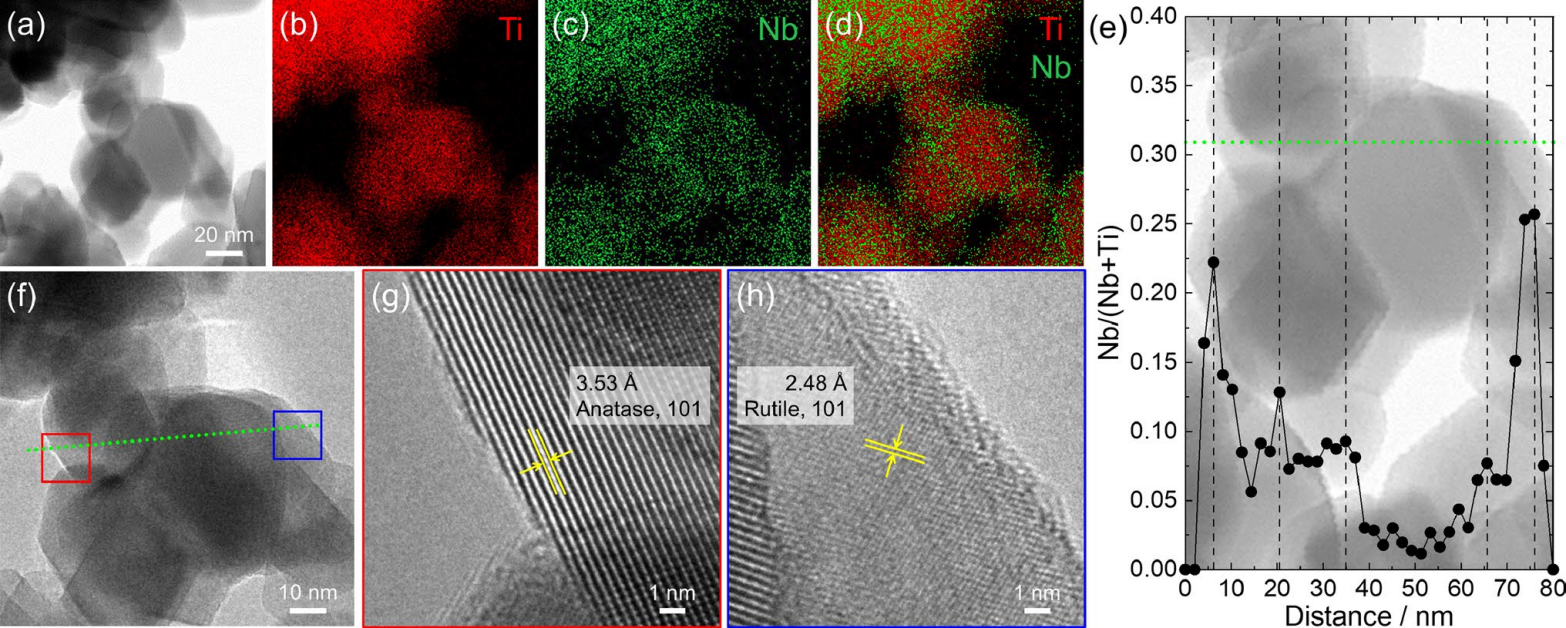
(**a**) STEM image, (**b**)–(**d**) STEM-EDS mapping images, and (**e**) STEM-EDS line scanned profile of NTO-5–800. The dot line in (**e**) represents the scan points at the edge of the particles. (**f**)–(**h**) TEM images of NTO-5–800. In (**f**), red and blue squares are magnified area of (**g**) and (**h**) and green dots are STEM-EDS line scanned points shown in (**e**).

Further analysis was demonstrated using TEM and STEM-EDS. Figure 5a–d were STEM and STEM-EDS mapping images. Although Ti atoms were homogenously distributed in the individual particles, Nb atoms inhomogeneously distributed around the edges of the particles. Figure 5e is the result of line scanned STEM-EDS analysis. Nb/(Ti + Nb) clearly increased at the scan point of the particle edges. Figure 5f–h are TEM image of the location where STEM-EDS mapping and line scan were taken. The start and end points of STEM-EDS line scan were located at the nanoparticles of anatase and rutile, respectively. Therefore, Nb rich TiO_2_ (Nb/(Ti + Nb) ~ 0.25) were formed at the surface of both anatase and rutile nanoparticles. The changes of lattice fringes were analysed by fast Fourier transform method (Figure S4). Clear spots corresponding to anatase 101 and rutile 101 lattice fringes were obtained at the analysis area enough distant from the particle surface. On the other hand, the fast Fourier transform images prepared using the analysis area around surface showed blurred spots with broad ring patterns, which indicates segregation of Nb atoms at the surface made the lattice disordered. XPS, STEM-EDS, and TEM analyses indicate that gradient chemical composition was spontaneously developed; poorly Nb doped TiO_2_ at the core of the nanoparticles and highly Nb doped TiO_2_ at the surface of nanoparticles.

Optical properties were investigated using UV–Vis diffuse reflectance spectroscopy (Figure S5 and S6). Obtained diffuse reflectance spectra were converted to (*F*(*R*_∞_)h*ν*)^2^ versus h*ν* and (*F*(*R*_∞_)h*ν*)^1/2^ versus h*ν* plots for an evaluation of direct and indirect transitions, respectively. Direct band gap energies were decreased with increasing heat treatment temperature (Figure S6a). On the other hands, indirect band gap energies were slightly increased with increasing heat treatment temperature (Figure S6b). There is a rough linear relationship between *f*_R_ and the direct band gap energy (Figure S6c) and between Nb/(Ti + Nb) and indirect band gap energy (Figure S6d). These results indicate that rutile phase and niobium titanium oxide phases are dominant factor of direct and indirect band gaps, respectively. In fact, direct and indirect band gaps were approaching to corresponding band gaps; direct band gap of rutile phase is 3.0 eV^49^ and indirect band gap of TiNb_2_O_7_ phase is 3.1 eV^45^, respectively.

In summary, mechanochemical treatment using TiO_2_ nanoparticles and TiNb_2_O_7_ microparticles produced Nb doped TiO_2_ nanoparticles as a result of preferential amorphization of TiNb_2_O_7_ phase. Homogenously incorporated Nb atoms diffused to the surface of nanoparticles after heat treatment. Resultant nanoparticles of both anatase and rutile-based phase had gradient chemical composition of highly Nb doped TiO_2_ and poorly Nb doped TiO_2_.

### Photocatalytic performance

**Figure 6:**
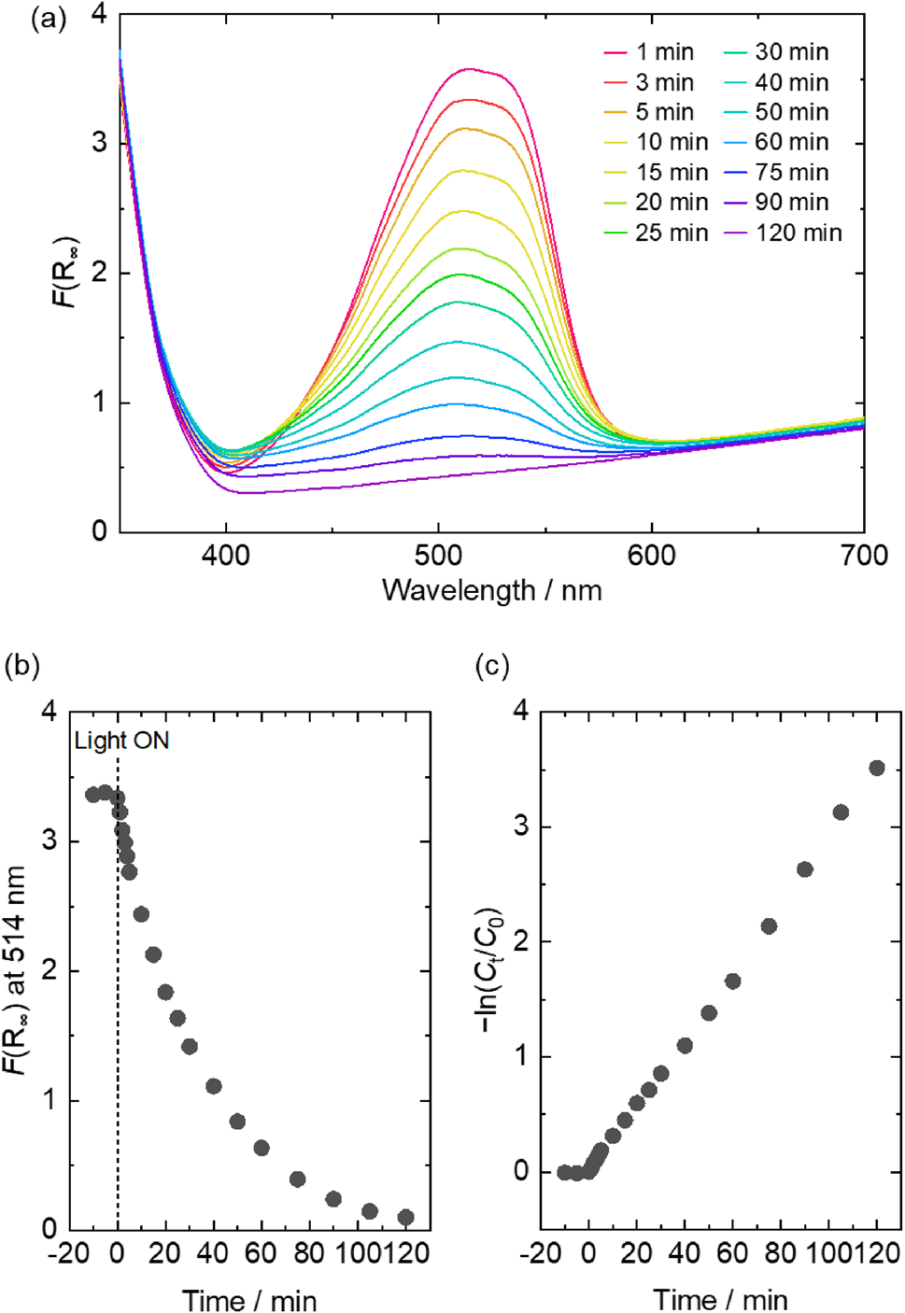
Decoloration of MO solution by using NTO-5–600 under VIS-light irradiation for appropriate time; (**a**) Kubelka–Munk plot, (**b**) time-dependent *F*(R_∞_) decrease, and (**c**) − ln(*C*_t_/*C*_0_) versus time plot.

**Figure 7:**
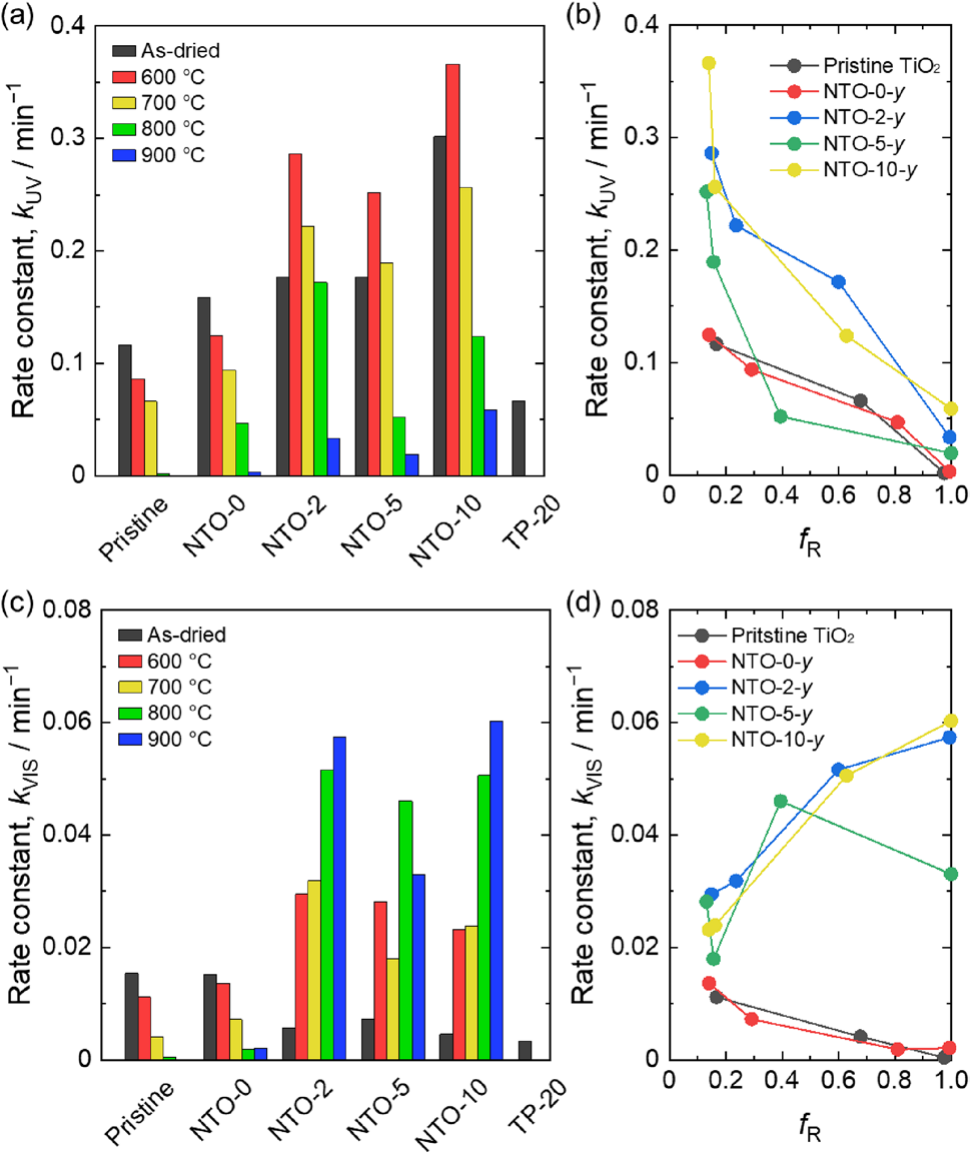
Rate constants, *k*_UV_ and *k*_VIS_, of photocatalytic decoloration of MO under irradiation of (**a**) UV-light and (**c**) VIS-light of P25, NTO-0-*y*, NTO-0-*y*, NTO-5-*y*, NTO-10-*y*, and TP-20. The plots of *f*_R_ versus (**b**) *k*_UV_ and (**d**) *k*_VIS_ of P25, NTO-0-*y*, NTO-0-*y*, NTO-5-*y*, NTO-10-*y*, and TP-20 (600 ≤ *y* ≤ 900).

The photocatalytic performances of mechanochemically prepared powders were evaluated by decoloration of methyl orange (MO) aqueous solution, which is widely employed to investigate the properties of photocatalysts. In addition to NTO-*x*–*y*, pristine TiO_2_ and homogeneously Nb incorporated TiO_2_ synthesized by thermal plasma treatment^32^ (Nb/(Ti + Nb) ~ 0.21, TP-20)) were tested as a control. Diffuse reflectance measurements were employed to trace decoloration of MO. Figure 6 shows typical results of MO decoloration. The peaks correspond to MO were clearly decreased with light irradiation time (Fig. 6a,b). The calculated −ln(*C*_t_/*C*_0_), where *C*_0_ and *C*_t_ were MO concentrations of initial and *t* min light irradiated solutions, were plotted against irradiation time (Fig. 6c). According to the following Eq. (3) for first-order reaction, the rate constants were determined;

3 $$- \ln \left( {C_{{\text{t}}} /C_{0} } \right) \, = k \cdot t$$

where *k* and *t* were rate constants (*k*_UV_ and *k*_VIS_ under UV- and VIS-light irradiation, respectively) and irradiation time. Figure 7a,c are summary of *k*_UV_ and *k*_VIS_ for all the tested samples. Both of *k*_UV_ and *k*_VIS_ of NTO-0-*y* were decreased with increasing heat treatment temperature same as TiO_2_. In the case of TiO_2_ and NTO-0-*y*, there was a relation between *f*_R_ and *k*_UV_/*k*_VIS_; the photocatalytic activity decreased with increasing *f*_R_ (Fig. 7b,d). Decrease of *k*_UV_ is due to decrease of UV-light active anatase phase. The trend of *k*_VIS_ is different from reported studies, in which heat treated TiO_2_ photocatalysts showed volcanic plot of VIS-light activity against *f*_R_^50–52^. The mechanism of photocatalytic activity in the case of anatase/rutile mixed phase was reported as follows; formation of heterojunction between anatase and rutile allows electrons transfer from rutile to anatase through interface that stabilizes charge separation and hinders charge recombination^53^. It is supposed that formed amorphous layers after mechanochemical treatment inhibit formation of anatase/rutile heterojunction, which disturbs electron transfer and results decrease of photocatalytic performance.

NTO-2-dry, NTO-5-dry, and NTO-10-dry showed significant increase of *k*_UV_ compared with NTO-0-dry and TiO_2_. Although mechanochemically synthesized samples showed increased *k*_UV_ with increasing Nb doping amount, TP-20 having largest amount of Nb dopant revealed lowest *k*_UV_. It is supposed that metastable doping by mechanochemical treatment promoted photocatalytic performance. The *k*_UV_ further improved after heat treatment at 600 °C only in the case of mechanochemically Nb doped samples, which indicates formed chemical compositional gradient enhanced photocatalytic reaction. The *k*_UV_ tend to decrease with increasing heat treatment temperature above 600 °C, *i*. *e*., increasing *f*_R_ as is shown in Fig. 7b. These results imply that Nb doped anatase nanoparticles having chemical compositional gradient structure are dominant component for an enhancement of UV-light photocatalytic performance. Although *k*_VIS_ of NTO-2-dry, NTO-5-dry, and NTO-10-dry were smaller than TiO_2_ and NTO-0-dry, those were drastically increased after heat treatment. Considering *k*_VIS_ increased with further increasing *f*_R_, it is supposed that Nb doped rutile nanoparticles gave significant enhancement of VIS-light photocatalytic performance. Surprisingly, NTO-2-800 showed significant high *k*_UV_ and *k*_VIS_ despite smaller specific surface area (3.21 m^2^/g) compared with pristine TiO_2_ (45.7 m^2^/g), NTO-2-dry (53.6 m^2^/g) and TP-20 (87.2 m^2^/g). It is reported that Cu nanocluster-grafted Nb-doped TiO_2_ showed VIS active photocatalytic performance owing to the energy level matching^54^. Considering these, formation of chemical compositional gradient structures will result energy level matching and lead excellent photocatalytic performance under both of UV- and VIS-light irradiation.

**Figure 8:**
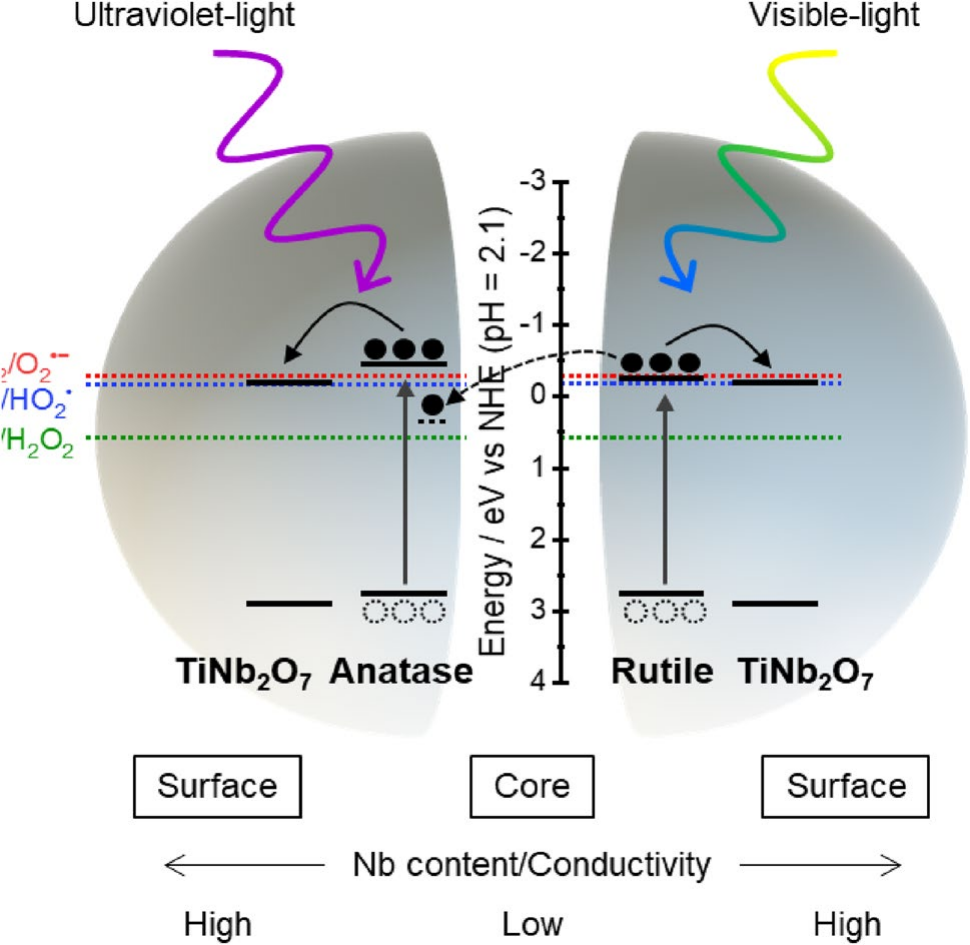
Schematic illustration of proposed mechanism of enhanced photocatalytic performance by Nb doped TiO_2_ nanoparticles with gradient chemical compositional structure. The reported energy levels were used^45,61,64^.

The effects of Nb doping on physical properties of TiO_2_ have been reported; with increasing amount of Nb dopant, band gap energy became large and electron conductivity enhanced^29,55–57^. In addition, Nb doping leads generation of Ti^3+^ improving photocatalytic activity^58,59^, which is not detected by XPS but detected by electron paramagnetic resonance technique^60^. Considering these reports, the hypothetic mechanism of mechanochemically prepared photocatalysts is summarized in Fig. 8. Electron–hole pair will generate at the poorly Nb doped TiO_2_ (core part of nanoparticles) due to their smaller band gap energy compared with highly Nb doped TiO_2_ (surface part of nanoparticles). Electrons will move to highly Nb doped TiO_2_ because of larger conductivity. In addition, electron will transfer from rutile to trapping site of anatase around heterointerface, which is reported to be 0.8 eV lower than the conduction band edge of anatase^61^. These processes will stabilize charge separation and retard charge recombination. Considering Nernstian shift of conduction bands of TiO_2_^62,63^, O_2_^−^ radical formation through single-electron reduction reaction may be minor reaction. HO_2_^−^ radical and H_2_O_2_ formed and attacked to MO molecules leading decoloration. The reported energy levels of valence band maximum and conduction band minimum of anatase, rutile, and TiNb_2_O_7_ (as a typical example of Nb-rich titanium oxides) consistent with our hypothesis^45,64^.

## Conclusion

Nb doped TiO_2_ were synthesized through mechanochemical reaction between TiO_2_ (rutile/anatase) nanoparticles and TiNb_2_O_7_ microparticles owing to the relatively lower elastic modulus of TiNb_2_O_7_. Homogeneously distributed Nb atoms tended to move to particle surface after high temperature heat treatment, which indicates initially formed Nb doped TiO_2_ was metastable phase. Resultant materials had gradient chemical compositional structure of poorly Nb doped TiO_2_ core part and highly Nb doped TiO_2_ surface part. It was found that the materials with gradient chemical compositional heterojunction structure showed enhanced photocatalytic activity under both of UV- and VIS-light irradiation. Obtained results in this study indicate metastable materials prepared by mechanochemical treatment will show unique functions which are different from materials prepared by conventional thermal processes. It is expected to enhance not only photocatalytic property but also other functionalities, such as catalytic and electrochemical activity, by design materials through mechanochemical treatment.

## Supplementary Information

Supplementary Information.
