# Supplementary material for: Spontaneously formed gradient chemical compositional structures of niobium doped titanium dioxide nanoparticles enhance ultraviolet- and visible-light photocatalytic performance
Source: Sci Rep. 2021 Jul 30;11:15236. doi: 10.1038/s41598-021-94512-x (PMC8324787; doi:10.1038/s41598-021-94512-x)
Supplement: Supplementary file 1 — Supplementary Information. [file 41598_2021_94512_MOESM1_ESM.docx]

# **Supplementary information**

**Spontaneously Formed Gradient Chemical Compositional Structures of Niobium Doped Titanium Dioxide Nanoparticles Enhance Ultraviolet- and Visible-Light Photocatalytic Performance**

*Naoki Tarutani^a,b,c,*^, Ryuma Kato^b^, Tetsuo Uchikoshi^c,d^, Takamasa Ishigaki^b,c*^*

*^a^ Applied Chemistry Program, Graduate School of Advanced Science and Engineering, Hiroshima University, 1-4-1 Kagamiyama, Higashi-Hiroshima, Hiroshima 739-8527, Japan.*

*^b^ Department of Chemical Science and Technology, Faculty of Bioscience and Applied Chemistry, Hosei University, 3-7-2 Kajino-cho, Koganei, Tokyo 184-8584, Japan.*

*^c^ Research Center for Micro-Nano Technology, Hosei University, 3-11-15 Midori-cho, Koganei, Tokyo 184-0003, Japan.*

*^d^ Research Center for Functional Materials, National Institute for Materials Science, 1-2-1, Sengen, Tsukuba, 305-0047, Japan*

**Figure S1.** XRD patterns of as dried and heat-treated precipitates synthesized by hydrolysis and condensation reaction of TTIP and NPE. Simulated pattern was prepared using referential TiNb_2_O_7_ (JCPDS 00-072-0116).

**
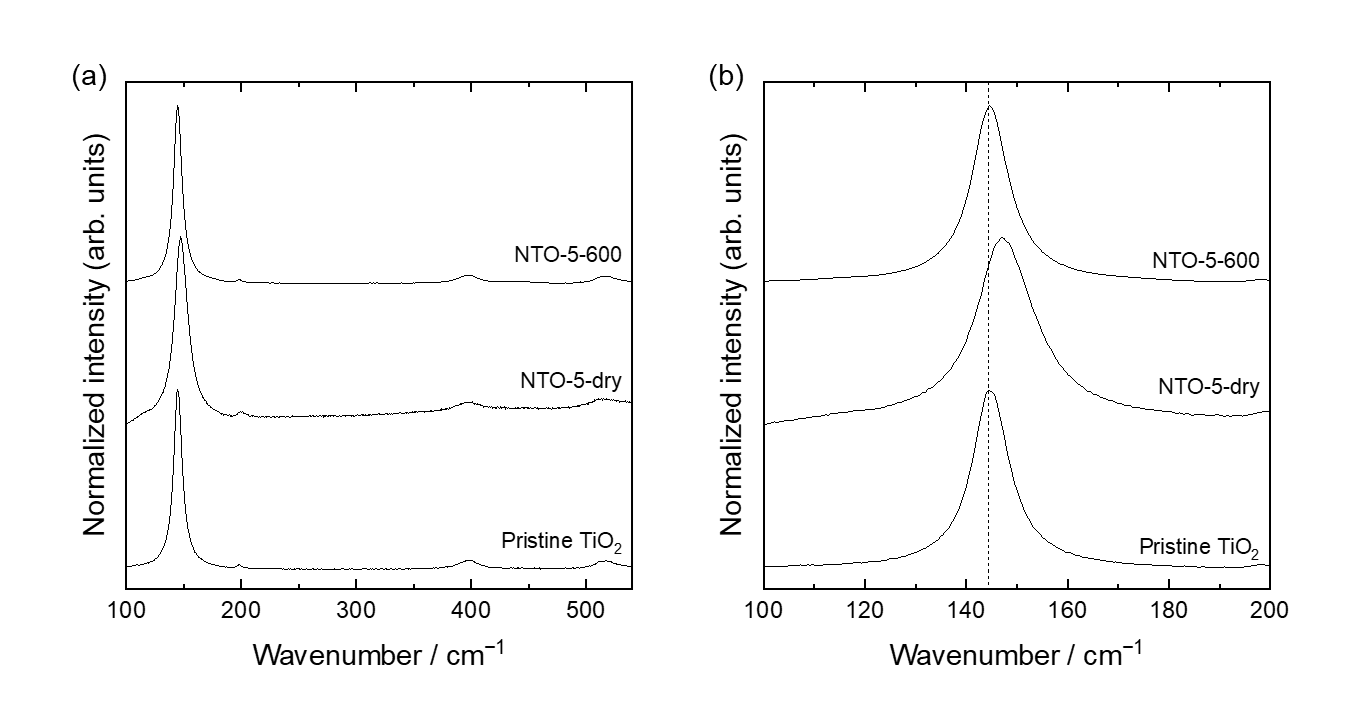
**

**Figure S2.** (a)(b) Raman spectra of pristine TiO_2_, NTO-5-dry, and NTO-5-600.

**Table S1.** Molar ratio calculated from SEM-EDS and XPS spectra.

| Sample ID | Nb/(Ti+Nb) | | |
| --- | --- | --- | --- |
|  | Nominal | SEM-EDS | XPS |
| NTO-2-dry | 0.020 | 0.021 | 0.023 |
| NTO-5-dry | 0.050 | 0.042 | 0.063 |
| NTO-10-dry | 0.10 | 0.11 | 0.099 |

**
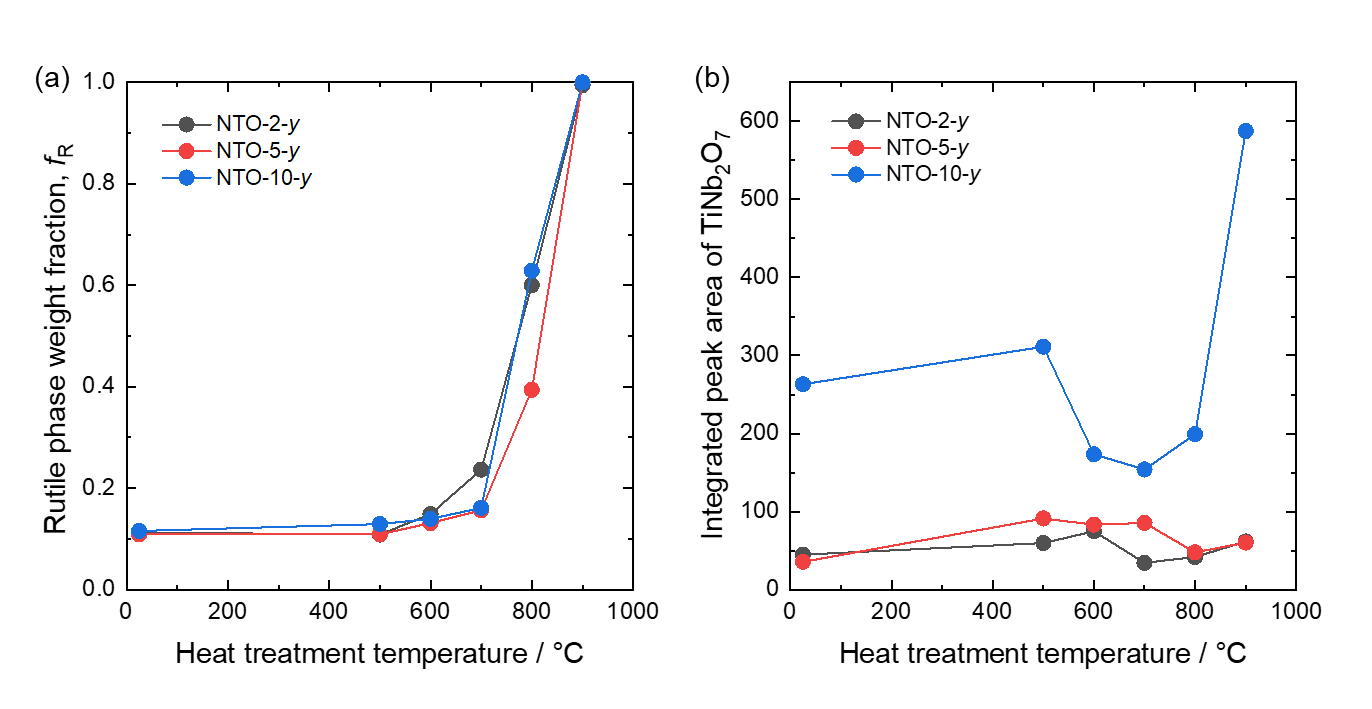
**

**Figure S3.** (a) Rutile phase weight fraction change and (b) integrated peak area of TiNb_2_O_7_ 20-5 diffraction peak change depending on heat treatment temperature.


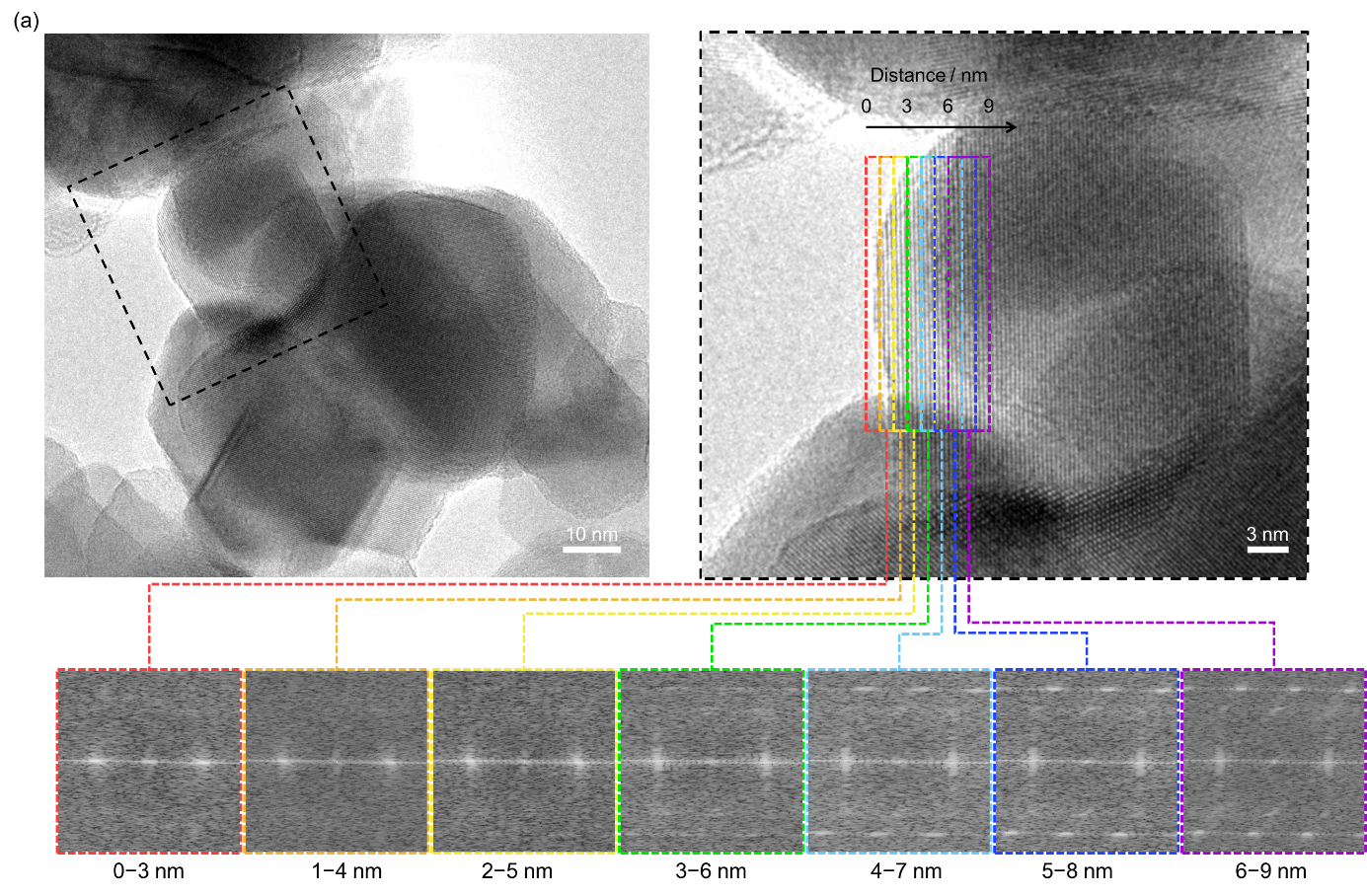


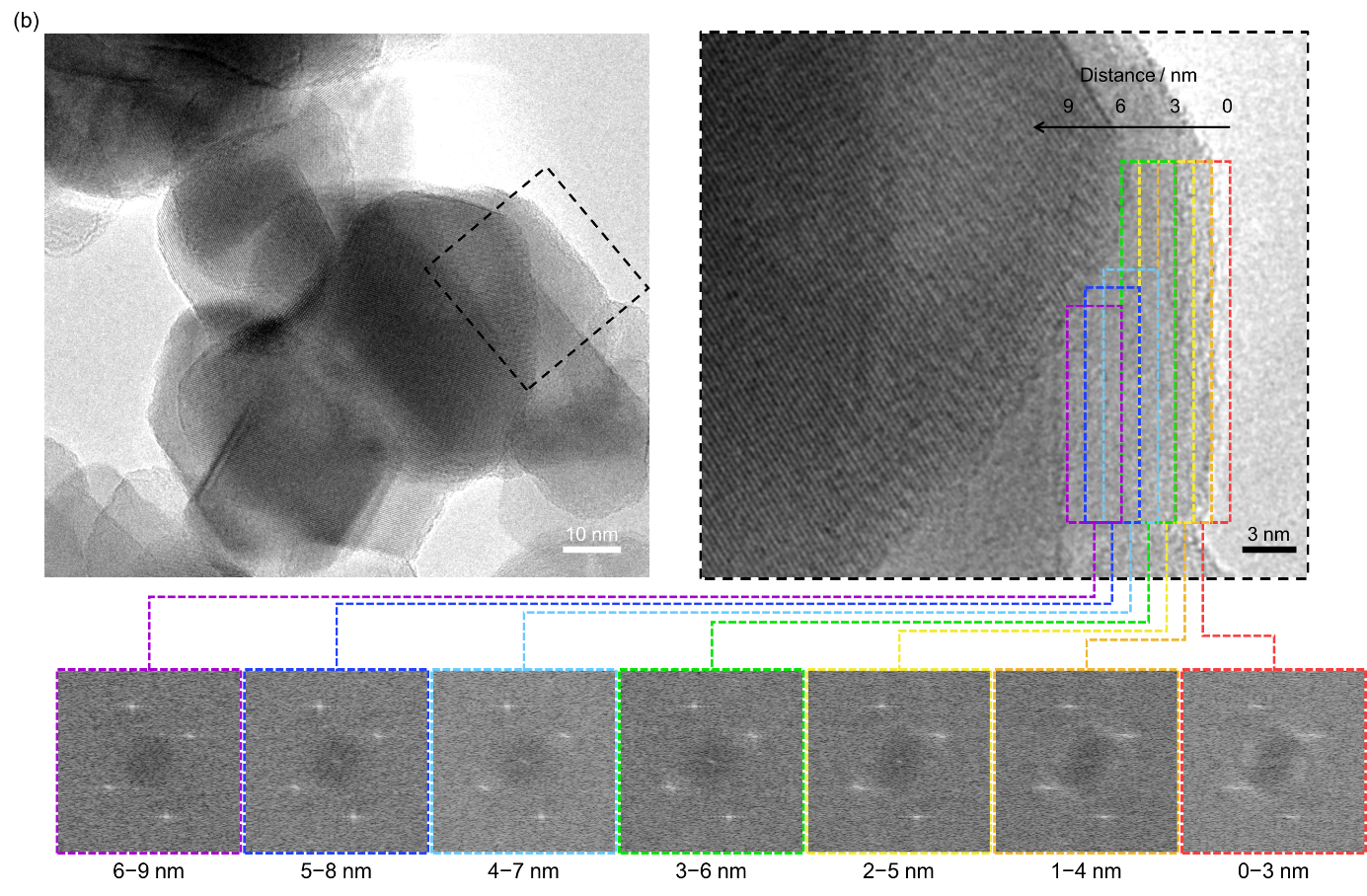


**Figure S4.** TEM images and area selected FFT images of (a) anatase and (b) rutile nanoparticles. The analyzed areas are (a) 3×20 nm^2^ and (b) 3×14−3×20 nm^2^.

**Figure S5.** Diffuse reflectance spectra of NTO-5-dry, NTO-5-700, NTO-5-800, and NTO-5-900


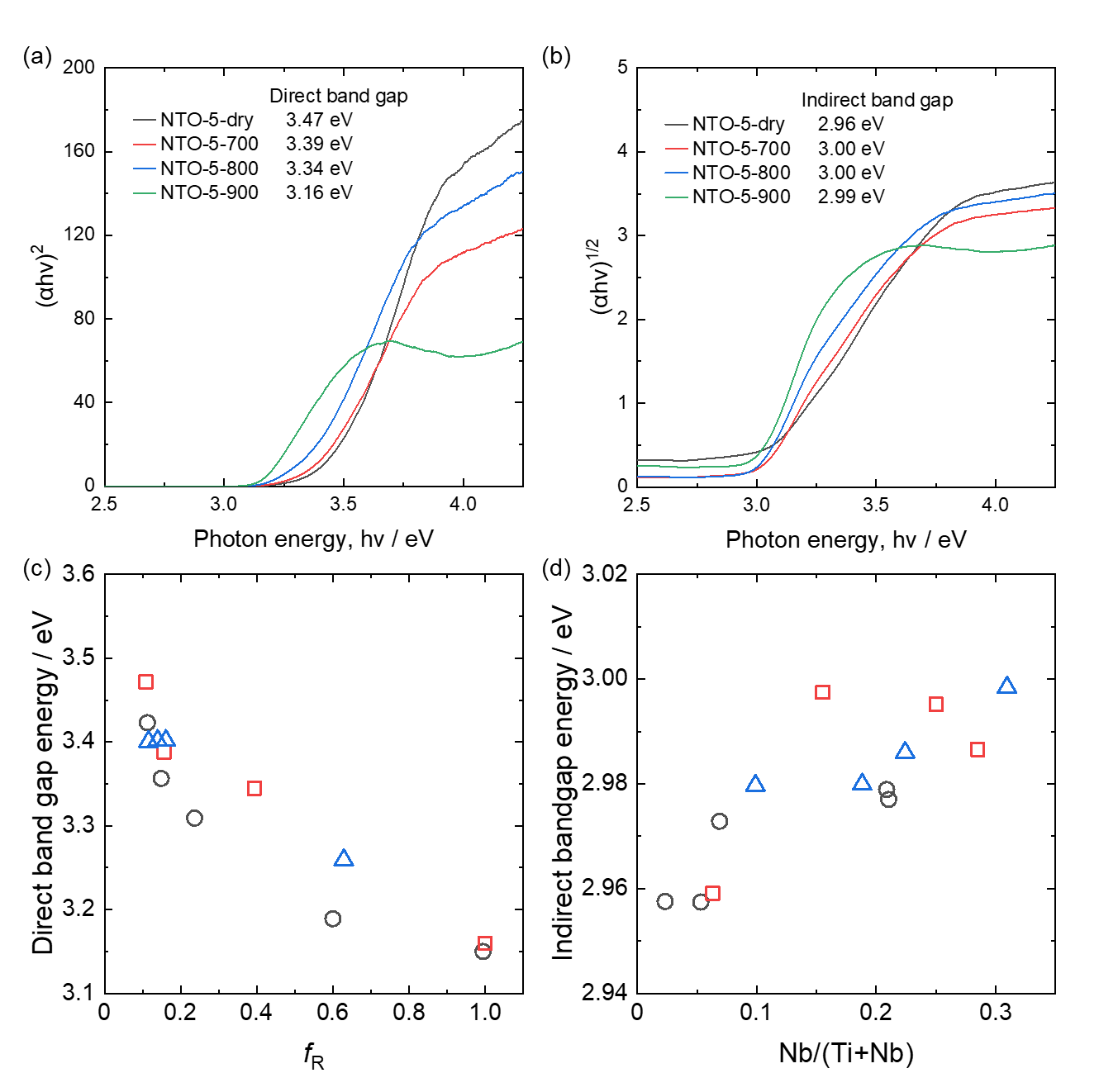


**Figure S6.** Kubelka-Munk plots of NTO-5-*y*; (a) (*F*(*R*_∞_)*hν*)^2^ vs *hν* and (b) (*F*(*R*_∞_)*hν*)^1/2^ vs *hν*. (c) *f*_R_-dependent change of direct band gap and (d) Nb/(Ti+Nb)-dependent change of indirect band gap of NTO-2-*y* (black circle), NTO-5-*y* (red square), and NTO-10-*y* (blue triangle).
